# Compulsory Returns of All Cases of Pulmonary Tuberculosis the First Step Towards Public Measures of Prevention and Treatment

**Published:** 1904-12

**Authors:** Eugene R. Corson

**Affiliations:** Savannah, Ga.


### COMPULSORY RETURNS OF ALL CASES OF PULMONARY TUBERCULOSIS THE FIRST STEP TOWARDS PUBLIC MEASURES OF PREVENTION AND TREATMENT.

BY EUGENE R. CORSON, M.D.,

SAVANNAH, GA.

To give an idea of the prevalence of tuberculosis in Savannah, I show a table which I have drawn up from the annual reports of the health office of this city from 1893 to 1903, inclusive. It gives the number of deaths per annum for white and colored, and their respective ratios per thousand and the average ratio per thousand each race. So far as the number of deaths per annum is concerned the figures are fairly reliable. Of course, in estimating the ratio per thousand, the population of the city is, in a measure, guessed at, and the resulting figures are only approximations. Still, I think the figures are as reliable as those from other cities, and are perhaps sufficiently accurate to use as comparisons.

Again, it is evident that pulmonary tuberculosis, while representing the most important factor in tuberculosis in general, still is far from being the whole question at issue. It must be remembered, too, that a certain number of cases fail to be reported from honest mistakes in diagnosis, as well as from intentional efforts to conceal the ugly fact. Such returns as "undefined," "continued fever," malarial fever" and "typhoid fever," are sometimes blinds as well as honest mistakes, for it is not very uncommon among the colored for deaths to occur unattended by a physician.

DEATHS FROM PULMONARY TUBERCULOSIS IN THE CITY OF SAVANNAH FROM 1893 TO 1903, INCLUSIVE.

| YEAR | No. of Deaths Among Whites | No. of Deaths Among Colored | Ratio per 1,000 (Whites) | Ratio per 1,000 (Colored) | Total |
|---|---|---|---|---|---|
| 1893 | 49 | 108 | 1.7 | 4.9 | 157 |
| 1894 | 54 | 123 | 1.8 | 5.3 | 177 |
| 1895 | 49 | 136 | 1.6 | 5.5 | 185 |
| 1896 | 80 | 132 | 2.6 | 5.8 | 212 |
| 1897 | 42 | 115 | 1.4 | 4.6 | 157 |
| 1898 | 45 | 113 | 1.5 | 4.5 | 158 |
| 1899 | 41 | 120 | 1.3 | 4.8 | 161 |
| 1900 | 80 | 130 | 2.6 | 5.2 | 210 |
| 1901 | 52 | 141 | 1.9 | 5.2 | 193 |
| 1902 | 55 | 154 | 1.8 | 4.5 | 209 |
| 1903 | 52 | 160 | 1.6 | 4.9 | 212 |

Average ratio per 1,000 for whites, 1.8.
Average ratio per 1,000 for colored, 5.01.

To attempt to ferret out other phases of tuberculosis, to obtain a more complete picture from such returns as marasmus, phthisis abdominal, meningitis, chronic bronchitis, consumption of the bowels, etc., seemed to me a hopeless task, and really quite unnecessary from what I assume to be the great object of this commission, namely, the consideration of those measures of public sanitation in regard to tuberculosis, which the whole scientific world now realizes should become a part of the State's jurisdiction. We all realize what a wide swath this disease cuts. Pulmonary tuberculosis is the great menace, and whatever measures are adopted by the profession as a body, or by the State or the general government, to meet this greatest of evils, covers its other manifestations.

The figures I have given in the table will represent fairly well, I think, the state of affairs existing along our Southern seaboard among the urban population. In the country and smaller towns they are less menacing. From a rather minute study of the general mortality of the two races in the South, which I made in 1903 when I wrote my paper on the negro,* I judge that these figures

*The Vital Equation of the Colored Race and Its Future in the United States. The Wilder Quarter Century Book, 1893.

represent the problem before us through a very large area of the South; that they show the conditions existing since the Civil War, and that these same conditions will exist for some time to come.

So far as the whites are concerned, the figures are somewhat better than those for New York City, for example, where the ratio of deaths trom tuberculosis is a little over two per cent. per thousand of population. The figures for the colored are comparable with the high mortality of Vienna and some other cities in Germany and Scotland.

It is claimed by many that we have no right to separate the two races in our estimate of the general health of our city, that the colored can be compared with the lower element in the Northern cities. It has been demonstrated, however, that the colored are especially susceptible, and that their mortality exceeds that of the worst element North. From the standpoint of general sanitation, however, the position taken is a correct one. There being no dearth of the contagiousness and infectiousness of tuberculosis the South is confronted by a great danger, which will have to be fairly and squarely faced. Every civilized country has waked up to it. The problem is being met in two ways: By measures to prevent the spread of the disease and by public efforts to care for those unable to care for themselves. What has been done in New York City, for example, is the admiration of two continents. And we shall have to follow as best we can. To Germany belongs the honor of having first shown the wonderful results of institutions devoted exclusively to the care of consumptives. Now, other countries, including our own, are following in her footsteps. Our own general government can show great results from the hospital in New Mexico. The States of Massachusetts, Pennsylvania and New York have done much in the same direction. We shall all have to follow as soon as possible.

The first step for us to take is undoubtedly the compulsory return of every case of tuberculosis which is recognized, and that compulsion should be as rigid as now exists in the case of diphtheria, scarlet fever and smallpox. And this step must be taken before we attempt any other public measures in the direction of the care and treatment of the great multitude of sufferers from this greatest of all plagues. And this step again will be of the greatest benefit in many ways. It will compel more careful examinations and diagnoses; it will lead to an earlier detection of the disease; it will give us more accurate returns; it will encourage more than anything else those measures of prevention and treatment which the individual and the State must combine in carrying out. Any amount of argument could be brought forward to show the necessity of this preliminary step, but it is unnecessary. All that need be said is that all the contagious and infectious diseases which are of sufficient importance to demand State recognition start from this basis. The State must know how great the danger is, and this can come only from proper returns. The live consumptive is more dangerous to the community than the dead one, and the earlier we detect his trouble the better for him and the better for the State.

And in no state of the Union is this question of such vital importance as it is in Georgia. Our State heads the lists in the number of colored. According to the census of 1900, we have at our door 1,034,813 colored, a race whose susceptibility to tuberculosis is more than twice as great as our own, and if there is any meaning in contagion and infection we have our hands full. For the State to attempt to grasp at once the situation in its entirety is impossible. Not only is the money lacking to enable it to carry out all the necessary measures, all the acquirements in the case, but it will first have to carefully and vigorously study all the phases of the problem, and this will mean time and perhaps a great deal of time. But it can make a beginning and that beginning, to my mind, should be compulsory returns of all cases of pulmonary tuberculosis, giving a reasonable time for its recognition.

If this commission can carry this point it will have accomplished a great work in itself and will have laid the foundation for a greater work in the future. It will not require a greater outlay of money than the State should be fully equal to. It will be the first great stimulus towards the proper consideration of those public measures of the prevention and treatment of the disease among the poorer classes, of which we have so large a contingent. We may talk as much as we please of the necessity of public sanatoria and public measures of prevention and treatment, but it will avail nothing unless we start from the beginning, and that beginning must be proper returns showing us the extent of the problem before us. We must know not only how many have died last year from tuberculosis, but how many foci of infection and contagion we have to handle this year. We must bell the cat. The extent and the rigorousness of this compulsory return is a question of serious thought and investigation, and will have to be settled. In a measure the problem has been successfully worked out in New York City and it will be comparatively an easy task to follow in the same footsteps, modifying certain features to meet the different conditions which confront us in the South. The stimulus which this step will give towards an earlier and more careful diagnosis of the disease will prove of the greatest benefit to all concerned. Thanks to Koch and his followers, the detection of the disease has been so easy that it requires no greater skill than that necessary for an insurance examination of urine. A few reagents, cover-glasses and slides, and a sufficiently good microscope, which I imagine now is not uncommon even in the country, do the work in fifteen or twenty minutes. A physician who cannot so equip himself and learn to make his own examination had better get out of the profession. He has no business practicing medicine. In the cities there is even less excuse, for the health office and the hospital should be prepared to make the necessary examination. In New York this is all done for the busy practitioner. The value of correct and early returns to the city, county and State is great enough to induce all health boards to undertake the work. It will be but a small item in the sanitary measures, which will be necessary if this disease is brought under municipal or State control.

The report of a case of pulmonary tuberculosis carries with it those instructions to the patient which aids him in the treatment of his case and in the prevention of any further spread of the disease from him as a focus. What this has done in New York City has been forcibly shown by Dr. Hermann M. Biggs. It is quite unnecessary that I should give the figures which speak so eloquently of the success of his work in public sanitation. The literature on the subject is free to all.

What New York and other States have done we can do, though I am aware the problem is a much more difficult one for us in the South for various reasons. Our work will be slow, but we can make it sure. The mere fact that this commission has been formed is sufficient pledge that the State stands ready to carry out all measures that the profession will decide upon as demanded by the great question at issue. The time is ripe and the occasion is pressing.

